# A Facile Method to In-Situ Synthesize Porous Ni_2_GeO_4_ Nano-Sheets on Nickel Foam as Advanced Anode Electrodes for Li-Ion Batteries

**DOI:** 10.3390/nano6110218

**Published:** 2016-11-19

**Authors:** Delong Ma, Xiaomin Shi, Anming Hu

**Affiliations:** 1 Institute of Laser Engineering, Beijing University of Technology, No. 100 Pingle Yuan, Chaoyao District, Beijing 100124, China; madl@bjut.edu.cn (D.M.); xiaomin501386@163.com (X.S.); 2 Department of Mechanical, Aerospace and Biomedical Engineering, University of Tennessee Knoxville, 1512 Middle Drive, Knoxville, TN 37996, USA

**Keywords:** Ni_2_GeO_4_, porous nanosheets, Ni foam, Li-ion batteries, Self-standing

## Abstract

A strategy for growth of porous Ni_2_GeO_4_ nanosheets on conductive nickel (Ni) foam with robust adhesion as a high-performance electrode for Li-ion batteries is proposed and realized, through a facile two-step method. It involves the low temperature hydro-thermal synthesis of bimetallic (Ni, Ge) hydroxide nanosheets precursor on Ni foam substrates and subsequent thermal transformation to porous Ni_2_GeO_4_ nanosheets. The as-prepared Ni_2_GeO_4_ nanosheets possess many interparticle mesopores with a size range from 5 to 15 nm. The hierarchical structure of porous Ni_2_GeO_4_ nanosheets supported by Ni foam promises fast electron and ion transport, large electroactive surface area, and excellent structural stability. The efficacy of the specially designed structure is demonstrated by the superior electrochemical performance of the generated Ni_2_GeO_4_ nanosheets including a high capacity of 1.8 mA·h·cm^−2^ at a current density of 50 μA·cm^−2^, good cycle stability, and high power capability at room temperature. Because of simple conditions, this fabrication strategy may be easily extended to other mixed metal oxides (M_*x*_GeO_*y*_).

## 1. Introduction

Mixed metal oxides, M_*x*_GeO_*y*_ (M = Zn, Cu, Cd, Co, Ge and In), as one of the most important families of functional inorganic materials, have numerous applications in the field of catalysts and optical devices, due to their abundant physical and chemical properties [1,2,3,4,5,6,7]. For example, Zn_2_GeO_4_ (with a band gap of 2.6–2.8 eV) can be used as a visible-light-sensitive photo-catalyst [5]. Ni_2_GeO_4_ loading with Pt nanoparticles exhibited high catalytic activity for CO oxidation [8]. However, the synthesis of such mixed metal oxides is still very challenging by wet chemistry routes because of the high valence state of Ge^4+^ and high stability of GeO_2_ that make GeO_3_^2−^ only exist in an alkaline environment. So the most successful work with respect to the synthesis of M_*x*_GeO_*y*_ (except for Zn_2_GeO_4_) is based on a solid-chemistry strategy using Na_2_GeO_3_ as the precursors. Furthermore, MO_*x*_GeO_2_ is a more common final product than pure-phase M_*x*_GeO_*y*_. Because most of the transition metal ions have strong metallicities, the single-component transition metal oxides are very stable. Therefore, it is important and urgent to develop an aqueous and controllable synthesis of M_*x*_GeO_*y*_.

Ni_2_GeO_4_ is an ideal candidate as high-performance anode materials for Li-ion batteries (LIBs). Because it contains the cheaper metal elements of Ni, which can reduce the content of deficient and expensive Ge, the overall cost will thus be effectively cut [9,10,11,12]. Furthermore, in theory, the Ni can be used as both a buffering and conductive agent during cycling, which is beneficial for battery performance [13,14,15]. However, there are limited studies focusing on Ni_2_GeO_4_-based anodes materials for Li-ion batteries [16,17].

Recently, nanostructures engineering has become a primary and popular strategy to improve the performance of batteries, as nanostructures have several advantages over their bulk counterparts [18,19,20,21,22,23]. First, a nanostructure provides a short transport path which can shorten the Li ions diffusion time. Second, the nanostructure may induce large numbers of electroactive sites into electrodes, which is beneficial to the high current rate performance [20]. More importantly, with nanostructures, the strain can be significantly reduced during lithiation and dilithiation process; thus, the nanostructure preserves the structural integrity of the electrode and leads to a more stable cycle performance [21,22]. However, most electrodes are commonly binder-enriched electrodes made by traditional slurry-coating technique for electrochemical evaluation. It is evitable that the organic binder will decrease the portion of the electroactive surface and block the contact of electrode with electrolyte. Moreover, the binder will seriously decrease the electrical conductivity of the electrode materials. Therefore, to achieve superior electrochemical performance, it is highly desirable to directly disperse and wire up electroactive mesoporous to an underlying conductive substrate. By this method, the tedious fabrication process of the electrode can be avoided. More importantly, electroactive materials with large naked surface and good electrical conductivity can have direct contact with both the electrolyte and the substrate for high efficiency energy storage [18,19].

Based on the aforementioned considerations, we developed a facile two-step strategy to grow porous Ni_2_GeO_4_ nanosheets on Ni foam with robust adhesion. The hybrid structure of Ni_2_GeO_4_@Ni foam was then directly used as an anode for Li-ion batteries at room temperature. Remarkably, the as-prepared 3D hybrid structure of Ni foam supported porous Ni_2_GeO_4_ exhibited good cycling stability. This evidenced Ni_2_GeO_4_@Ni foam as a promising electrode for Li-ion batteries.

## 2. Materials and Methods 

Germanium oxide (GeO_2_, AR), Ni foam (2 mm thick, 420 g·m^−2^, Changsha Lyrun New Material Co. Ltd., Changsha, China), Concentrated Ammonia (NH_4_OH, AR, Beijing Chemical Works, Beijing, China), HCl (AR, Beijing Chemical Works, Beijing, China). All reagents were used as received without any further purification.

X-ray diffraction (XRD) patterns were collected on Bruker D8 Advance Powder X-ray diffractometer (Karlsruhe, Germany) using Cu Kα radiation. The scanning electron microscopy (SEM) was performed by using a field emission scanning electron microscopy (FESEM, HITACHI, S-4800, Tokyo, Japan). Transmission electron microscopy (TEM), high-resolution transmission electron microscopy (HRTEM), and energy dispersive X-ray spectroscopy were conducted with a JEM-2100 electron microscope (JEOL, Tokyo, Japan). X-ray photoelectron spectroscopy (XPS) analysis was carried on an ESCALAB MK II X-ray photoelectron spectrometer (VG scientific instrument co., Landon, UK).

The electrodes were cut into disks (with a diameter of 8 mm) before transferring into an Argon-filled glove box. Coin cells (CR2025) were laboratory-assembled using Li metal as the counter electrode, Celgard 2400 membrane as the separator and 1 M LiPF_6_ in ethylene carbonate (EC)/dimethyl carbonate (DMC) (EC/DMC, 1:1 wt %) as the electrolyte. The galvanostatic charge-discharge tests were carried out on a Land Battery Measurement System (Land, Wuhan, China). Cyclic voltammetry (CV) was performed using a VMP3 Electrochemical Workstation (Bio-logic Inc., Grenoble, France).

In a typical procedure, 100 mg of GeO_2_ was added in 30 mL water and 100 μL ammonium hydroxide was dissolved into water to form transparent solutions. Then the pH of the solution was adjusted to 7.1 with 1 M HCl. After that, a piece of Ni foam (washed by ethanol) was added into the mixed solution. Then the mixed solution was transferred into a stainless steel autoclave with a Teflon liner of 50 mL capacity and heated in an oven at 120 °C for 7 h. After the autoclave had cooled naturally to room temperature, the Ni foam was rinsed with distilled water and ethanol alternately. Finally, Ni_2_GeO_4_ on Ni foam was obtained by annealing the precursor in N_2_ at a ramp rate of 4 °C·min^−1^ and holding at temperatures of 700 °C for 1 h.

## 3. Results and Discussion

### 3.1. Electrode Synthesis and Microstructure Characterization

In our synthesis strategy, two steps are involved: mixed metal (Ni, Ge)O_*x*_OH_*y*_ precursors in situ grown on Ni foam followed by a calcination process in N_2_ to form Ni_2_GeO_4_. First, the NiO (metallic Ni on the surface of Ni foam can react with O_2_ in air to form NiO) on the surface of Ni foam reacted with NH^4+^ to form Ni(NH_3_)_6_^2+^ which is the nickel resource during the reaction. According to this method, it can avoid rapid formation of Ni(OH)_2_ precipitation due to the high concentration of Ni^2+^ in the solution, which is unfavorable for in situ growth of (Ni, Ge)O_*x*_OH_*y*_. Then, the generation of Ni(NH_3_)_6_^2+^ results in the uniform precipitation of mixed (Ni, Ge)O_*x*_OH_*y*_ on the Ni foam surface by reacting with GeO_3_^2−^ and OH^−^ in the solution. The whole process may comprise of electrochemical reactions as follows:

(1) GeO_2_ + NH_4_OH ➔ GeO_3_^2−^ + NH_4_^+^ + H_2_O

(2) NiO + NH^4+^ ➔ Ni(NH_3_)_6_^2+^ + H_2_O

(3) Ni(NH_3_)_6_^2+^ + GeO_3_^2−^ + OH^−^ ➔ (Ni, Ge)O_*x*_OH_*y*_ + NH_3_

Subsequently, the formed precursors were thermally transformed to black Ni_2_GeO_4_ supported on the Ni foam. The dehydration reaction can be described as follows:

(4) (Ni, Ge)O_*x*_OH_*y*_ ➔ Ni_2_GeO_4_ + H_2_O

**Figure 1:**
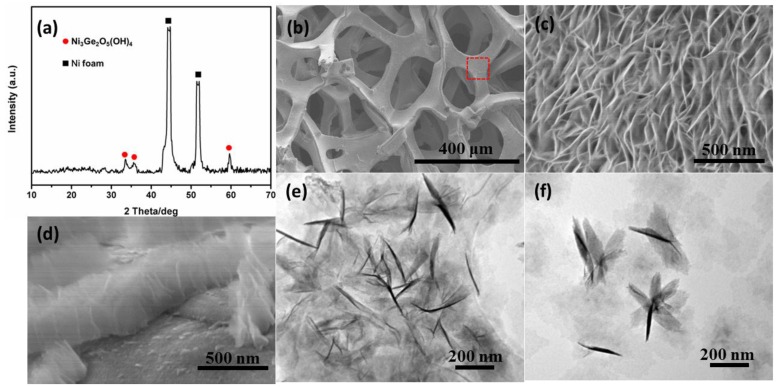
(**a**) X-ray diffraction (XRD) patterns, (**b**–**d**) scanning electron microscopy (SEM), and (**e**,**f**) transmission electron microscopy (TEM) images of as-prepared precursor.

The morphology and structure of the as-prepared products were investigated by different characterization techniques. As shown in Figure 1a, the XRD patterns confirm that one of the constituents for the precursor is Ni_3_Ge_2_O_5_(OH)_4_. The peaks at 2θ = 33.44°, 35.47° and 59.47° can be well indexed with the (132), (206) and (330) of Ni_3_Ge_2_O_5_(OH)_4_ reflections (JCPDS No. 11-0097), respectively. However, the molar ratio between Ni and Ge in Ni_3_Ge_2_O_5_(OH)_4_ precursor (3:2) is lower than that in the final products Ni_2_GeO_4_ (2:1). It is possible that this difference arises from the partial constituent of the precursor (Ni(OH)_2_ with poor crystallinity) [8]. Therefore, the precursor has been estimated to be (Ni_3_Ge_2_O_5_(OH)_4_)·(Ni(OH)_2_), which is in accord with the Ni/Ge molar ratio of Ni_2_GeO_4_. The energy dispersive X-ray spectroscopy (EDX) analysis has proved that Ni and Ge element exactly co-exist in the precursor (as shown in Figure S1). Figure 1b shows a representative low-magnification SEM image of the precursor supported on a Ni foam substrate. It can be found that the Ni foam remains in a 3D grid structure with hierarchical macro-porosity after the hydrothermal reaction. To further reveal its microstructure, Figure 1c shows a high magnification top-view SEM image of Figure 1b (the region marked). Evidently, the precursor is a layer of uniform nanosheets vertically aligned on the surface of the Ni foam. The thickness of the nanosheet layer is about 400 nm (as shown in the Figure 1d). TEM measurements were carried out to further investigate the structure of as-prepared precursor. This further confirms the nanosheet structure of precursor, as shown in Figure 1e. It can be found that nanosheets exhibit transparent silk-like morphology, indicating the ultrathin nature, which is analogous to graphene. Some nanosheets crosswise assemble to form a petal structure, as shown in Figure 1f. Furthermore, we found that the as-obtained precursors are also highly sensitive to the pH of the mixed solution. Figure S2a,b shows SEM images of the precursor without controlling the pH value (the pH value is about 8.7). Instead of nanosheets, there are only some nanoparticles on the surface of the Ni foam. In addition, the NH^4+^ and GeO_3_^2−^ are also necessary to form nanosheets. As shown in Figure S2c,d, there are also some nanoparticles on the surface of Ni foam after hydrothermal reaction without adding GeO_2_. Analogously, the nanosheet does not form during the hydrothermal reaction without adding NH_4_OH, as shown in Figure S2e. Furthermore, the reaction time also affects the morphology of nanosheets. Figure S3 shows SEM images of samples obtained at different stages of the reaction, 3 and 12 h respectively. It can be found that nanosheets become more and more compact and thick along with the increase of reaction time. When the reaction time is 7 h (shown in Figure 1c), the density and thickness of nanosheet are the most suitable. So we chose 7 h as the reaction time. It should be noted that the nanosheet precursor can also grow in situ on other conductive substrates, such as Cu foil (Figure S4a–d) and stainless steel wire (Figure S4e–h). These results indicate that the current synthesis is a general method, which may be used to prepare other mixed metal oxides.

**Figure 2:**
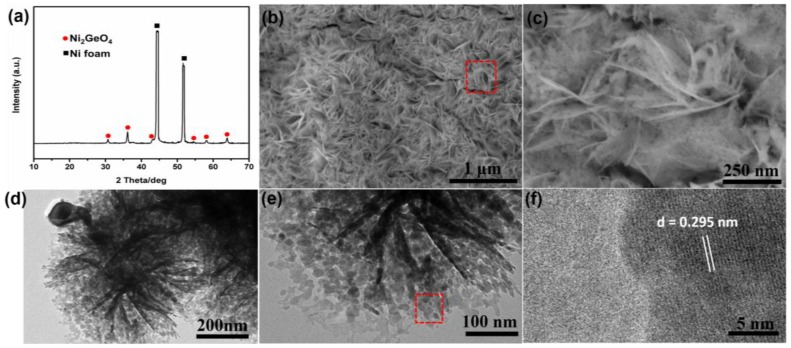
(**a**) XRD patterns, (**b**,**c**) SEM, (**d**,**e**) TEM, and (**f**) high-resolution TEM (HRTEM) images of as-prepared Ni_2_GeO_4_.

In order to obtain the pure-phase Ni_2_GeO_4_ nanocrystals, the precursors were sintered at 700 °C for 1 h under N_2_ atmosphere. The phase-transformation is quite obvious after heat treatment, as shown in the XRD patterns of Figure 2a. The peaks corresponding to Ni_3_Ge_2_O_5_(OH)_4_ disappear completely. The six new peaks at 2θ = 30.69°, 36.14°, 43.28°, 54.59°, 58.14° and 63.87° appear, which are well indexed with the (220), (311), (400), (422), (511) and (440) of Ni_2_GeO_4_ reflections (JCPDS No. 10-0266), respectively. After conversion into Ni_2_GeO_4_, the basic morphology of the samples is conserved without calcination-induced significant alterations, as shown in Figure 2b,c. These as-formed nanosheets are intertwined with each other, which creates loose porous nanostructures with abundant open space for volume expansion and electroactive surface sites. TEM measurements were carried out to further investigate the structure of the as-synthesized samples. The TEM images in Figure 2d,e illustrates that the product retained hierarchical flower-like nanostructures. Due to the much larger lateral size than the thickness, the morphologies of bending, curling, and crumpling are clearly observed. The dark strips are generally the folded edges or wrinkles of the nanosheets. Moreover, it can be clearly seen that numerous inter-particle mesopores with a size ranging from 5 to 15 nm are generated after the heat treatment owing to the loss of high contents of OH^−^ groups in the precursors. The lattice spacing of nanoparticles is about 0.295 nm in the HRTEM images (Figure 2f), which corresponds well with the characteristic (220) planes of fluorite phase Ni_2_GeO_4_. It is well known that the mesoporous structures in nanosheets are important to facilitate the mass transport of electrolytes within the electrodes for fast charging/discharging. The porous structure will also greatly increase the electrode/electrolyte contact area and buffering of the volume change during cycling, and thus further enhance the electrochemical performance.

**Figure 3:**
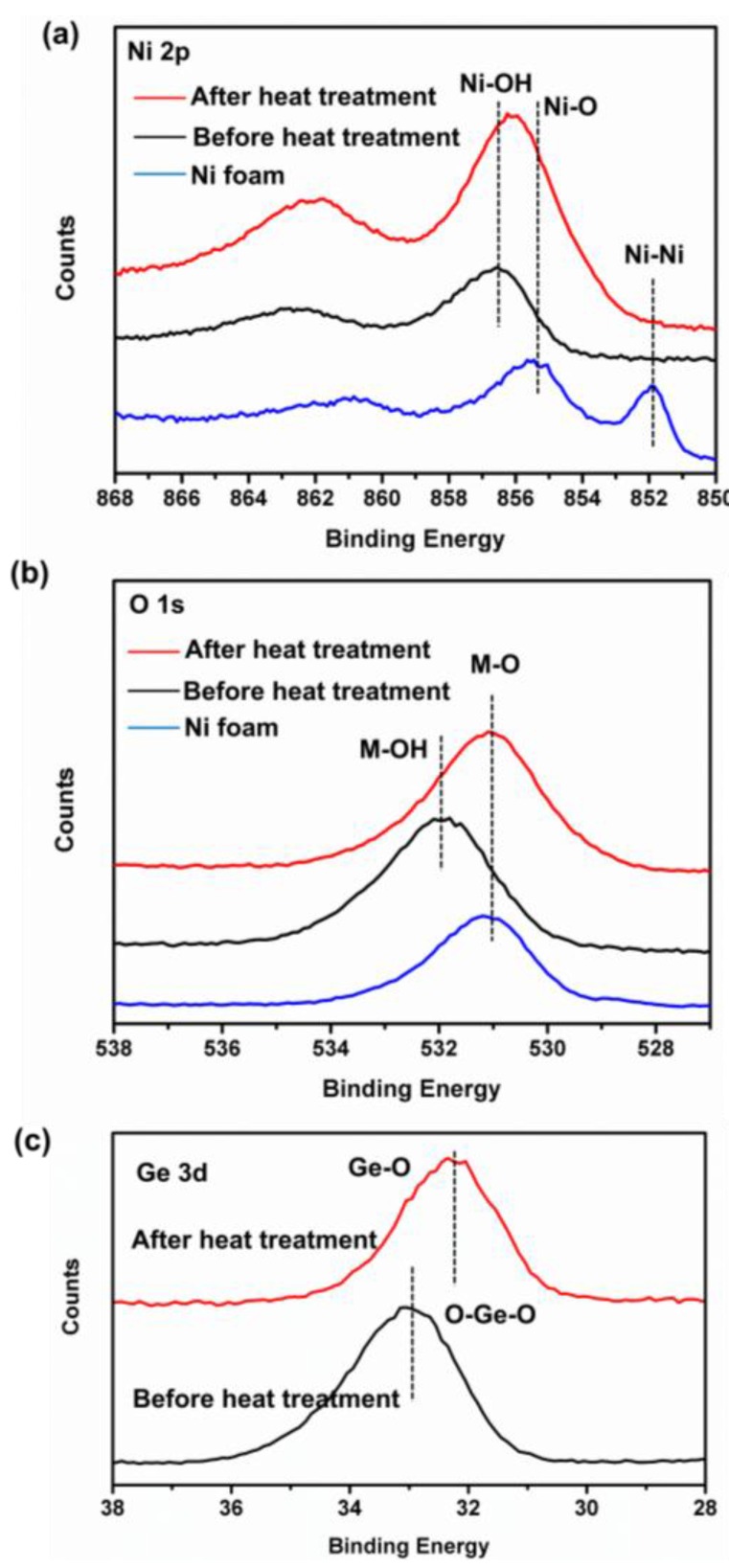
X-ray photoelectron spectroscopy (XPS) spectra of (**a**) Ni 2p, (**b**) O 1s and (**c**) Ge 3d for the as-prepared samples.

The more detailed elemental composition and the oxidation state of the as-prepared Ni_2_GeO_4_ are further characterized by X-ray photoelectron (XPS) measurements. Figure 3a shows the Ni 2p emission spectrum. The Ni 2p at 851.8 eV is typical of metal-oxygen (NiO) bonds, indicating that NiO forms on the surface of Ni foam. After growing the precursor on the Ni foam, the Ni 2p shifts to 856.5 eV, because of the formation of Ni–OH bonds [24], which is in accord with the result of XRD (Figure 1a). After heat treatment, the Ni 2p shifts to 856 eV. This is due to the loss of OH^−^ groups and forming of Ni–O bonds at high temperature. It should be noted that the binding energy of Ni 2p in Ni_2_GeO_4_ is higher than that in NiO, which is characteristic of Ni^3+^. Figure 3b shows the O 1s emission spectrum. It can be found that the changed trend of O 1s is similar to that of Ni 2p, indicating a process of dehydration during heat treatment [25]. Figure 3c shows the Ge 3d emission spectrum of as-prepared samples. The binding energy become lower after heat treatment [26], indicating the forming of Ge^2+^. It may be caused by the redox reaction between Ge^4+^ and Ni^2+^ at high temperature. These data show that the surface of the as-prepared Ni_2_GeO_4_ has a composition containing Ge^4+^, Ge^2+^, Ni^2+^ and Ni^3+^.

### 3.2. Cell Assembly and Electrochemical Performance

**Figure 4:**
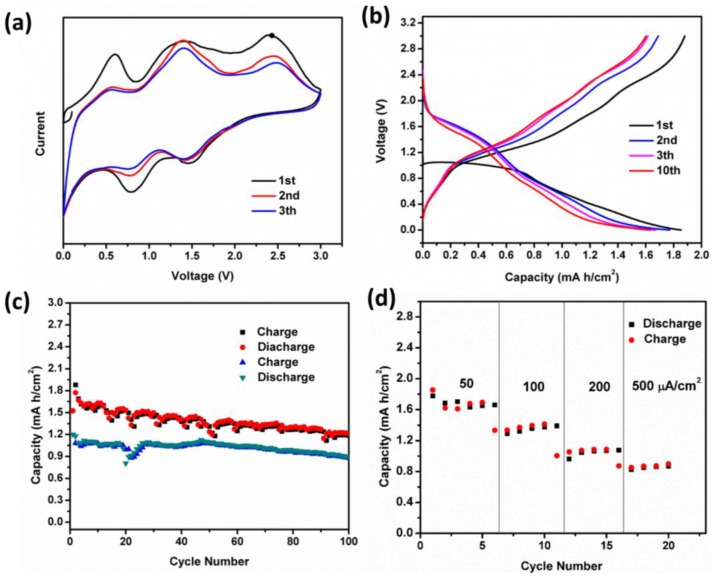
(**a**) Representative cyclic voltammetry (CV) curves of Ni_2_GeO_4_ porous nanosheets for the first 3 cycles at a scan rate of 0.5 mV·s^−1^ between 0.01 V and 3 V; (**b**) Voltage profiles of Ni_2_GeO_4_ porous nanosheets at a current density of 50 μA·h·cm^−2^; (**c**) Cycling performance of Ni_2_GeO_4_ porous nanosheets at a current density of 50 and 200 μA·h·cm^−2^; (**d**) Rate performance of Ni_2_GeO_4_ porous nanosheets.

To highlight the merits of the unique architecture, we directly use the hybrid structure of porous Ni_2_GeO_4_ nanosheets supported on Ni foam as a self-standing anode for Li-ion batteries. Coin cells with metallic Li counter electrode are assembled, directly applying the Ni foam support Ni_2_GeO_4_ nanosheets as an electrode. Galvanostatic discharge-charge technique is employed to evaluate the electrochemical performance at room temperature. To identify the lithiation mechanism of Ni_2_GeO_4_, cyclic voltammetry (CV) was first performed. Figure 4a shows the CV scans of as-prepared samples in the voltage window of 0.01–3 V at a scan rate of 0.2 mV·s^−1^. In the first cathodic scan, three main reduction peaks were observed. The remarkable cathodic peak located at around 1.5 V is ascribed to the insertion of Li^+^ into Ni_2_GeO_4_ to form Li_*x*_Ni_2_GeO_4_ [27,28,29]. The cathodic peak located at around 0.75 V is ascribed to the decomposition of Li_*x*_Ni_2_GeO_4_ into Ni, Ge and Li_2_O and the formation of the solid electrolyte interface (SEI) film [30,31]. The sharp peak starting at about 0.25 V indicates the Li-Ge alloying reactions [30]. In the anodic scan process, a broad peak center at 0.55 V can be observed, which is assigned to the delithiation of Li-Ge alloys. It is noted that a peak located at 1.4 V presents the reoxidation of Ni and Ge. The peak located at 2.4 V may correspond to the reforming of Ni_2_GeO_4_, which is different from the CV curves of Zn_2_GeO_4_ [4]. This may be correlated to the high valence state Ni^3+^. However, further research is needed to confirm these analyses. More state-of the-art characterization methods, such as electron energy loss spectroscopy (EELS) spectra recorded in TEM mode with a spread beam, would be helpful to provide direct evidence.

Figure 4b shows the discharge-charge voltage profiles cycled under a current density of 50 μA·cm^−2^ over the voltage range of 0.01–3 V vs. Li^+^/Li. The initial discharge and charge areal capacities are 1.85 and 1.9 mA·h·cm^−2^, respectively. It should be noted that the charge capacity is higher than discharge, which is due to the oxidation of Ni on the surface of Ni foam at high voltage. After the first cycle, the discharge profile can be divided into three parts: the initially sloped area between 2.0 and 1.0 V stems from the insertion of Li^+^ into Ni_2_GeO_4_, because of a typical solid solution reaction [32,33,34]. This is followed by another sloped area from 1 to 0.4 V, which is due to the electrochemical decomposition of Ni_2_GeO_4_ to Ni and Ge through a conversion reaction. Finally, the sloped area from 0.4 to 0.01 V is assigned to the delithiation of Li-Ge alloys, according to the results of CV test. It can be found that the first discharge profile is not in keeping with the CV curve and the following discharge profiles. This is a universal phenomenon for metallic oxide [35,36,37,38]. It is worth noting that there is a discharge sloped plateau at about 1.5 V (after first cycle) with a capacity 0.7 mA·h·cm^−2^. So Ni_2_GeO_4_ is also a promising cathode material with high capacity for Li-ion batteries.

The cycling performance of Ni_2_GeO_4_ porous nanosheets is shown in Figure 4c, which exhibits its excellent cyclability with high capacity. At the current density of 50 μA·cm^−2^, the reversible areal capacity is 1.9 mA·h·cm^−2^ in the first cycle and remains at 1.25 mA·h·cm^−2^ after 100 cycles. Even at the high current density of 200 μA·cm^−2^, an appealing cycle performance of 0.9 mA·h·cm^−2^ after 100 cycles can be obtained. Notably, the Ni_2_GeO_4_ porous nanosheets still shows high Li^+^ storage and excellent cycling stability even at a very high rate (Figure 4d). For testing, the cell was discharged/charged at various current densities from 50 to 500 μA·cm^−2^ each for five cycles. The reversible areal capacities are 1.7, 1.3, 0.9 and 0.8 mA·h·cm^−2^ at 50, 100, 200 and 500 μA·cm^−2^, respectively, exhibiting attractive rate performance.

## 4. Conclusions

In summary, an advanced three-dimensional electrode was fabricated by growing porous Ni_2_GeO_4_ nanosheets on Ni foam for high-performance Li-ion batteries. The efficient two-step synthesis involves growing a bimetallic (Ni, Ge) hydroxide precursor on Ni foam, and subsequent thermal conversion into Ni_2_GeO_4_. The as-prepared Ni_2_GeO_4_ nanosheets possess numerous inter-particle mesopores with a size of 5 to 15 nm. Owing to these advantageous structural features, this hybrid electrode of porous Ni_2_GeO_4_ nanosheets supported on Ni foam are able to deliver high capacity with good cycling stability. The simple and effective synthesis strategies, coupled with excellent electrochemical performance, make Ni_2_GeO_4_ an attractive anode material for LIBs. Our work opens up the possibility of constructing advanced electrodes for mixed metal oxides (M_*x*_GeO_*y*_).

## Supplementary Materials

The following are available online at http://www.mdpi.com/2079-4991/6/11/218/s1.
