# Supplementary material for: A Facile Method to In-Situ Synthesize Porous Ni2GeO4 Nano-Sheets on Nickel Foam as Advanced Anode Electrodes for Li-Ion Batteries
Source: Nanomaterials (Basel). 2016 Nov 19;6(11):218. doi: 10.3390/nano6110218 (PMC5245747; doi:10.3390/nano6110218)
Supplement: Supplementary file 1 [file nanomaterials-06-00218-s001.pdf]

Supporting Information: A Facile Method to In-Situ Synthesize Porous Ni_2GeO_4 Nano-Sheets on Nickel Foam as Advanced Anode Electrodes for Li Ion Batteries

Delong Ma, Xiaomin Shi and Anming Hu

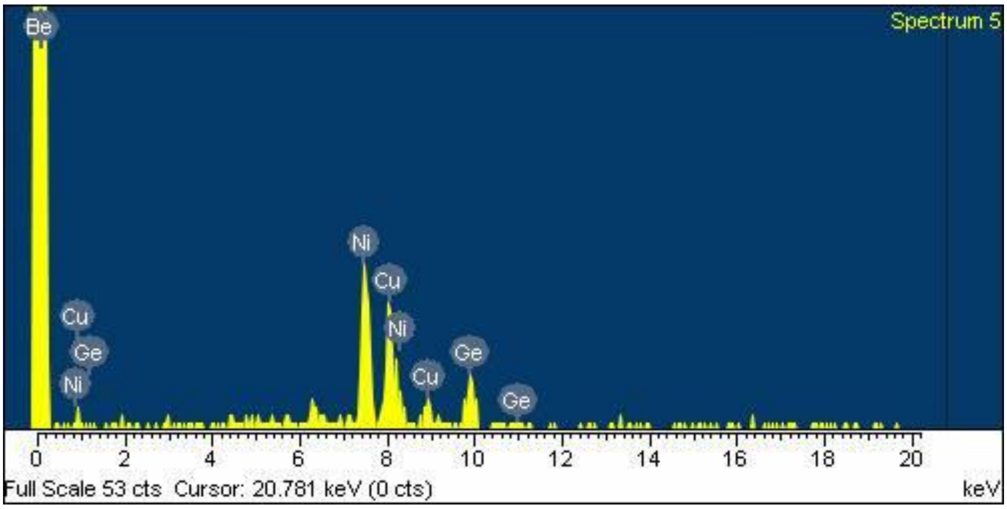

Figure S1. Energy dispersive X-ray spectroscopy (EDS) spectrum of the precursor.

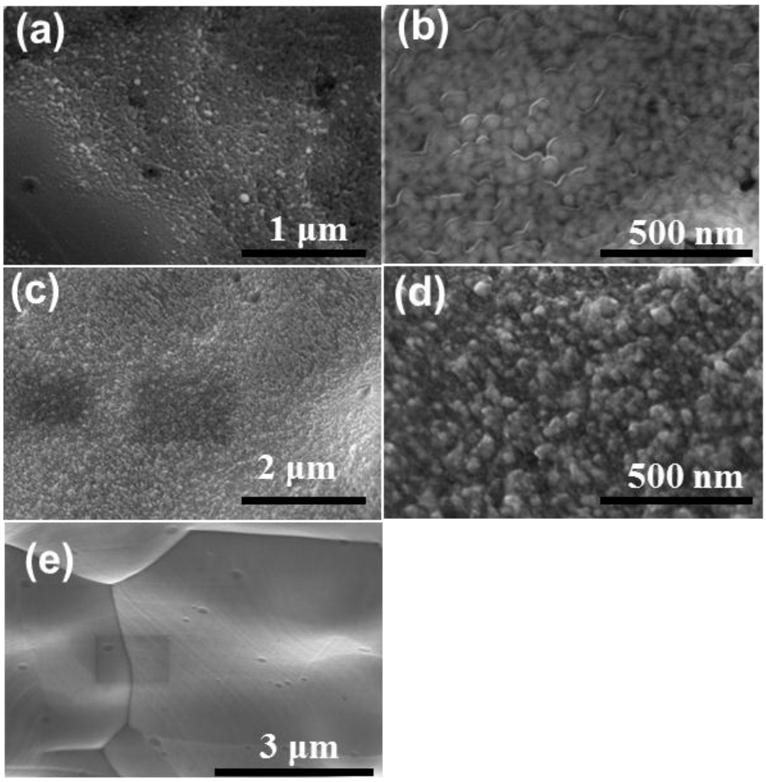

Figure S2. SEM images of as-prepared samples. (**a**,**b**) without controlling the pH, (**c**,**d**) without adding GeO_2, (**e**) without adding NH_4OH and GeO_2.

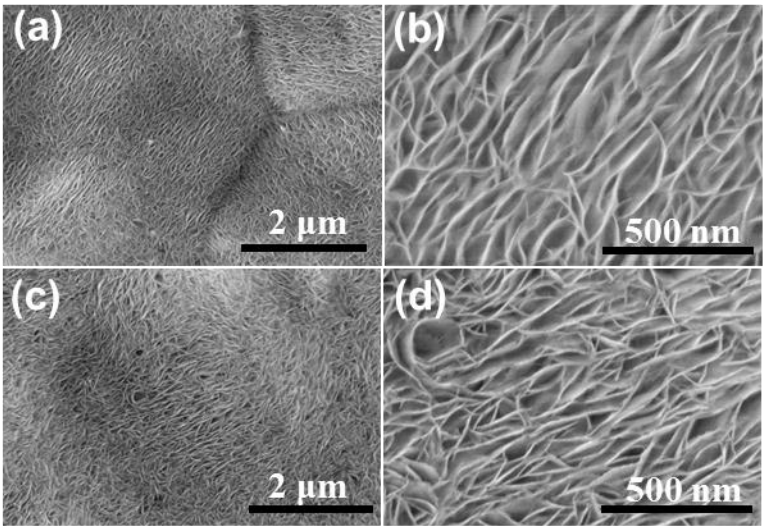

Figure S3. SEM images of as-prepared samples with different reaction time. (**a**,**b**) 3h and (**c**,**d**) 12 h.

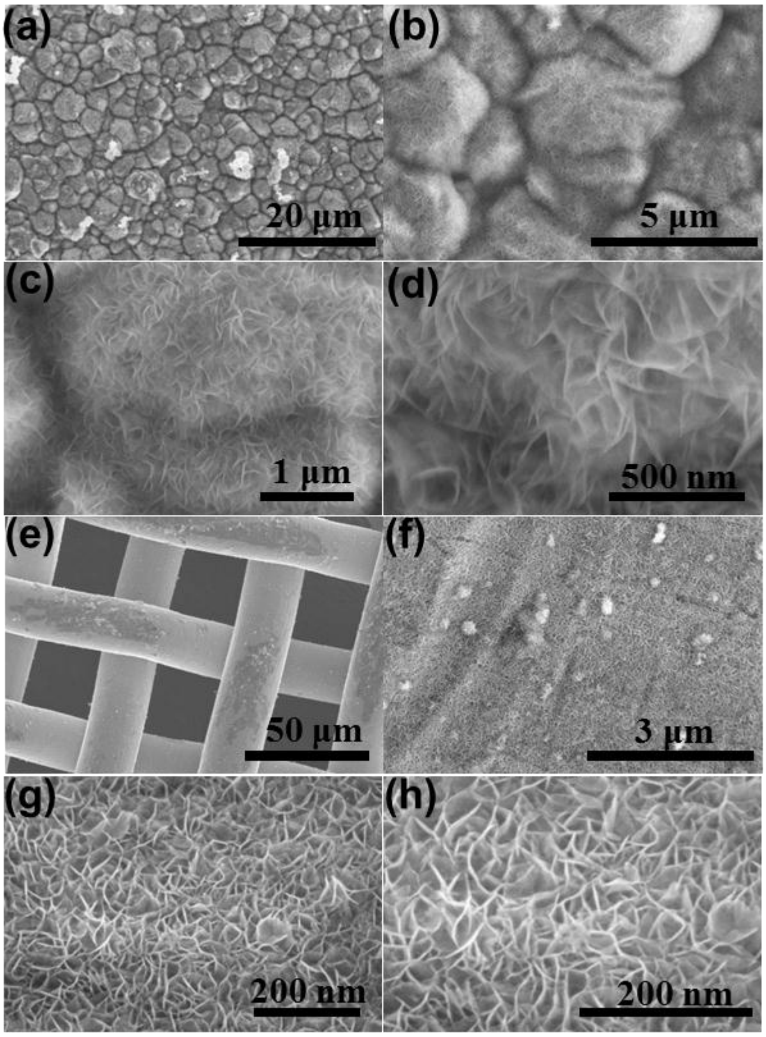

Figure S4. SEM images of as-prepared nano-sheet precursor at different conductive substrate. (**a**–**d**) on Cu foil and (**e**–**h**) on stainless steel wire.

© 2016 by the authors. Submitted for possible open access publication under the terms and conditions of the Creative Commons Attribution (CC-BY) license (http://creativecommons.org/licenses/by/4.0/).
